# A Composition of Phytonutrients for Glycemic and Weight Management

**DOI:** 10.3390/nu14183784

**Published:** 2022-09-14

**Authors:** Yasuyo Urasaki, Thuc T. Le

**Affiliations:** College of Pharmacy, Roseman University of Health Sciences, 10530 Discovery Drive, Las Vegas, NV 89135, USA

**Keywords:** berberine, cinnamaldehyde, curcumin, diabetes, glycemic control, insulin, nutrition intervention, obesity, serine/threonine protein kinase, weight management

## Abstract

Maintaining healthy body weight is an important component of any effective diabetes management plan. However, glycemic management using insulin generally leads to weight gain. In addition, weight loss medications prescribed for diabetes management are often associated with adverse side effects, which limit their long-term usage. Alternatively, nutrition intervention provides a safe, readily accessible, and inexpensive option for diabetes management. This study describes a composition of phytonutrients comprising berberine, cinnamaldehyde, and curcumin for glycemic and weight management. Functional complementarity between berberine, cinnamaldehyde, and curcumin provides an effective means to improve insulin sensitivity without increasing adiposity. In primary human omental preadipocytes, cinnamaldehyde and curcumin additively enhance insulin-stimulated activation of Akt2 and glucose uptake, whereas berberine inhibits de novo fatty acid biosynthesis and fat cell differentiation. In a diet-induced obesity murine model, a dietary supplement with berberine, cinnamaldehyde, and curcumin prevents weight gain, improves glucose tolerance, and reduces HbA1c, blood lipids, visceral adiposity, and liver steatosis. Collectively, the composition of phytonutrients comprising berberine, cinnamaldehyde, and curcumin protects against obesity and pre-diabetic conditions in a diet-induced obesity murine model. Safety and efficacy assessment of nutrition intervention using combined berberine, cinnamaldehyde, and curcumin for glycemic and weight management in future clinical trials are warranted.

## 1. Introduction

Diabetes mellitus is a major public health problem. Diabetes mellitus is a group of metabolic disorders characterized by chronic high blood glucose condition [1,2,3]. Two main types of diabetes mellitus are Type 1 diabetes or juvenile diabetes, and Type 2 diabetes, or adult-onset diabetes. Type 1 and Type 2 diabetes affect approximately 5% and 95% of all diabetic patients, respectively. Type 1 diabetes is a consequence of impaired insulin production due to a loss of pancreatic β cells. In contrast, Type 2 diabetes is a consequence of insulin resistance due to overweight and insufficient exercise. Signs and symptoms of both types of diabetes mellitus include increased thirst, frequent urination, extreme hunger, fatigue, blurred vision, and frequent infections. Long-term complications of diabetes mellitus include cardiovascular disease, stroke, neuropathy, nephropathy, retinopathy, foot ulcer, and cognitive impairment. In 2019, approximately 11.3% of the US population, or 37.3 million people of all ages, had diabetes [4]. The total cost of diabetes management in the US was estimated to be $327 billion in 2017 [5].

Managing diabetes generally involves glycemic and weight management. Managing Type 1 diabetes requires insulin injection to lower blood glucose levels [6]. In contrast, managing Type 2 diabetes involves glycemic and weight management [7]. The first line of defense against Type 2 diabetes includes maintaining a healthy body weight and regular physical exercise [8]. In addition, anti-diabetes and anti-obesity medications can be prescribed. On the one hand, anti-diabetes therapy using insulin lowers blood glucose levels but leads to weight gain. On the other hand, a number of FDA-approved anti-diabetes and anti-obesity therapeutics do not lead to weight gain but can be associated with adverse side effects, which may limit their long-term usage for glycemic and weight management [9].

Nutrition intervention is a viable alternative for diabetes management. Phytonutrients, or natural compounds found in plants, may have anti-obesity and anti-diabetes effects by modulating physiological pathways that regulate appetite, metabolism, and insulin sensitivity [10]. Consumption of phytonutrients is generally considered a safe, widely available, and inexpensive approach to managing obesity and diabetes. Notably, berberine, cinnamaldehyde, and curcumin are among the phytonutrients that are being evaluated clinically for their anti-diabetes effects [11,12,13,14]. Berberine and curcumin are phytonutrients isolated from the rhizomes of Chinese goldthread and turmeric plants, respectively [15,16]. Cinnamaldehyde is a phytonutrient isolated from the bark of cinnamon trees [13]. On the one hand, cinnamaldehyde and curcumin promote insulin-stimulated activation of Akt2, a serine/threonine protein kinase central to the insulin signaling pathway [17]. On the other hand, berberine improves systemic insulin sensitivity by lowering blood lipids and reducing body fat accumulation [12,18,19,20]. 

This study explores nutrition intervention for diabetes management via the improvement of insulin sensitivity without weight gain or adverse side effects. Specifically, a combination of phytonutrients comprising berberine, cinnamaldehyde, and curcumin for glycemic and weight management is examined. Primary human omental preadipocytes are used to investigate the interaction of berberine, cinnamaldehyde, curcumin, and insulin on the activation of Akt2, glucose uptake, and fat cell differentiation. In addition, a diet-induced obesity murine model is used to assess the efficacy of a dietary supplement comprising berberine, cinnamaldehyde, and curcumin for glycemic and weight management. 

## 2. Materials and Methods

### 2.1. Primary Human Omental Preadipocytes

Primary human preadipocytes were isolated from omental adipose tissues of an overweight (BMI > 30) and diabetic donor who was undergoing elective surgery (cat. no. OP-F-3, Zen-Bio, Durham, NC, USA). Primary human omental preadipocytes were maintained at 37 °C, and 5% CO_2_ in growth media comprising Minimum Essential Medium α (cat. no. 12571063, Thermo Fisher Scientific, Waltham, MA, USA) supplemented with 10% fetal bovine serum, 100 units/mL penicillin, and 100 µg/mL streptomycin.

### 2.2. Treatment Conditions

Existing growth media of primary human omental preadipocytes were replaced with new growth media premixed with treatment compounds and incubated at 37 °C and 5% CO_2_ for 30 min prior to the collection of total cell extracts. The final concentrations of treatment compounds were as follows: Insulin (100 nM, Humulin R, Eli Lilly, Indianapolis, IN, USA), berberine (2 µM, cat. no. 10006427, Cayman Chem, Ann Arbor, MI, USA), cinnamaldehyde (10 µM, cat. no. W228613, Sigma Aldrich, St. Louis, MO, USA), and curcumin (4 µM, cat. no. C7727, Sigma Aldrich). Half-maximal effective concentration of berberine to inhibit drug-induced adipogenesis of primary human omental preadipocytes was experimentally determined and chosen. Concentrations of cinnamaldehyde and curcumin were chosen based on two selection criteria: (a) An ability to affect post-translational modifications of Akt2 and enhance insulin-stimulated Akt2 phosphorylation at S474, and (b) an inability to suppress drug-induced adipogenesis of primary human omental preadipocytes. No effect on the viability of primary human omental preadipocytes was observed when treated with individual phytonutrients or with combinations of phytonutrients at chosen concentrations. 

### 2.3. Preparation of Total Cell Extracts

Approximately one million cells were incubated on ice for 10 min with 60 µL of lysis buffer (cat. no. 040-764, ProteinSimple, Santa Clara, CA, USA), sonicated 4 times for 5 s each, mixed by rotation for 2 h at 4 °C, and centrifuged at 12,000 rpm in an Eppendorf 5430R microfuge for 20 min at 4 °C. The supernatant was collected as the cell lysate. The total protein concentration in the cell lysate was determined with a Bradford protein assay and adjusted to a final concentration of 0.3 µg/µL with separation gradients (cat. no. Premix G2, pH 5–8, ProteinSimple, Santa Clara, CA, USA) for charge-based cIEF immunoassays.

### 2.4. Capillary Isoelectric Focusing Immunoassays

Cell lysates in separation gradients were loaded into 384-well assay plates (cat. no. 040-663, ProteinSimple), which were preloaded with primary and secondary antibodies and chemiluminescent substrates. Charge-based protein separation and detection in 96 individual capillaries were performed using the default protocols of the NanoPro 1000 system (ProteinSimple). HSP70 was used as the loading control. All cIEF immunoassays were performed in triplicate for each protein, and triplicate experiments were performed for each treatment condition, producing nine repeated measurements per protein analyte. 

### 2.5. Capillary Western Immunoassays

The total protein concentration in the cell lysate was determined with a Bradford protein assay and adjusted to a final concentration of 0.4 µg/µL with denaturing buffers (cat. no. PS-ST01EZ or PS-ST03EZ, ProteinSimple) for size-based Western immunoassays. Cell lysates in denaturing buffers were denatured at 95 °C for 5 min, and then transferred to assay plates (cat. no. SM-W004 or SM-W008, ProteinSimple) preloaded with blocking reagents, wash buffer, primary and secondary antibodies, and chemiluminescent substrates. Sized-based protein separation and detection in 24 capillaries were performed using the default protocols of the Jess system (ProteinSimple). β-Actin was used as a loading control. All capillary Western immunoassays were performed in triplicate for each protein, and duplicate experiments were performed for each treatment condition, producing six repeated measurements per protein analyte.

### 2.6. Data Analysis

Quantitation of protein abundance was performed using the Compass software from ProteinSimple and normalized with HSP70 for cIEF immunoassays or β-actin for capillary Western immunoassays.

### 2.7. Antibodies

Primary antibodies against Akt2 (cat. no. 3063, 1:50 dilution), p-Akt2 (S474) (cat. no. 8599, 1:50 dilution), PPARɣ (cat. no. 2443, 1:50 dilution), FASN (cat. no. 3189, 1:50 dilution), PLIN1 (cat. no. 9349, 1:20 dilution), and HSP70 (cat. no. 4872, 1:50 dilution) were purchased from Cell Signaling Technology (Danvers, MA, USA). Primary antibody against β-actin was purchased from R&D Systems (Minneapolis, MN, USA). Secondary antibodies (cat. no. 040-656 (1:100 dilution), 042-205 (no dilution), 042-206 (no dilution), 043-819 (no dilution), and 043-821 (no dilution)) were purchased from ProteinSimple.

### 2.8. Glucose Uptake Assays

Primary human omental preadipocytes were grown to 70% confluence in growth media, washed with phosphate-buffered saline, and replaced with glucose-free DMEM media. Following 2 h of incubation in glucose-free DMEM media, preadipocytes were treated with 100 µg/mL of 2-NBDG fluorescent glucose analog and insulin (100 nM) or phytonutrients (EC_50_) for 30 min. Preadipocytes were collected and analyzed using the Acuri C6 flow cytometer (BD Biosciences, San Jose, CA, USA) and the 485 nm excitation and 535 nm emission filters.

### 2.9. Adipogenesis Assays

Primary human omental preadipocytes were grown to confluence in growth media. At 2 days post-confluence, growth media were aspirated off the culture dishes and complete differentiation media were added. Complete differentiation media comprise DMEM with 18.5 mM glucose, HEPES (15 mM), NaHCO_3_ (25 mM), 100 units/mL penicillin, 100 µg/mL streptomycin, d-biotin (33 µM), pantothenate (17 µM), dexamethasone (100 nM), insulin (100 nM), rosiglitazone (1 µM), IBMX (0.5 mM), triiodothyronine (T3, 2 nM), and transferrin (10 µg/mL). On day three post-differentiation, complete differentiation media were replenished. On day seven post-differentiation, complete differentiation media were replaced with maintenance media. Maintenance media comprise DMEM, 100 units/mL penicillin, 100 µg/mL streptomycin, HEPES (15 mM), NaHCO_3_ (25 mM), d-biotin, pantothenate, insulin (10 nM), and dexamethasone (10 nM). Maintenance media were replenished ten days post-differentiation. Complete differentiation of preadipocytes into adipocytes was achieved on day fourteen post-differentiation. Phytonutrients were mixed in complete differentiation media from day 0 to day 6 post-differentiation. Phytonutrients were removed together with complete differentiation media on day 7 post-differentiation. Total cell extracts collected on day six post-differentiation were used to measure expression levels of PPARɣ, FASN, and PLIN1 using capillary Western immunoassays. Cell cultures were fixed and stained with hematoxylin, eosin, and Oil Red O on day fourteenth post-differentiation to assay for intracellular lipid droplet accumulation. 

### 2.10. Diet-Induced Obesity Murine Model 

C57BL/6J mice (male, ~10 weeks old, Jackson Lab, Bar Harbor, Maine) were divided into three groups: A group of 40 mice fed with a lean diet, a group of 40 mice fed with a high-fat diet, and a group comprising mice fed with a high-fat diet supplemented with a composition of phytonutrients comprising berberine, cinnamaldehyde, and curcumin at 1:1:1 weight ratio (F2 composition). The F2 composition was supplemented to the diet at 0.1% by weight, leading to an approximately daily dose of 200 mg/kg for mice, or approximately 16 mg/kg of human equivalent dose. The lean diet (cat. no. TD7001, Teklad Diets, Madison, WI, USA) comprised protein (25.2% by weight), carbohydrate (39.5% by weight), fat (4.4% by weight), and others (30.9% by weight, ash, fibers, others). The lean diet has 3 kcal/g, with 34% of kcal from protein, 53% of kcal from carbohydrates, and 13% of kcal from fat. The high-fat diet (cat. no. TD88137, Teklad Diets) comprised protein (17.3% by weight), carbohydrate (48.5% by weight), fat (21.2% by weight), and others (13% by weight, ash, fibers, others). The high-fat diet has 4.5 g/kcal, with 15.2% of kcal from protein, 42.7% of kcal from carbohydrates, and 42% of kcal from fat. Mice groups were placed on their respective diets in the form of ground pellets for 17 weeks. Glucose tolerance tests using standard protocols were performed in week 16. Terminal tissue and blood sample collection were performed in week 17. Collected liver and visceral adipose tissues were sent to IHC WORLD (Woodstock, MD, USA) for histopathology analysis. Commercial assay kits were used to analyze HbA1c (cat. no. LS-F36432, LS Bio, Seattle, WA, USA), triglyceride (cat. no. 65336, Abcam, Cambridge, MA, USA), cholesterol (cat. no. 65359, Abcam), HDL (cat. no. 65390, Abcam), and LDL (cat. no. 65390, Abcam) in collected blood samples. All animal studies were performed in conformity with the Public Health Service Policy on Humane Care and Use of Laboratory Animals and with the approval of the Animal Care and Use Committee at Roseman University of Health Sciences.

### 2.11. Statistical Analysis

Quantitative data were presented as the mean values ± standard deviations. Statistical analysis was performed using Microsoft Excel software (Microsoft, Redmond, WA, USA). Statistical significance was calculated using Student’s paired *t*-test with two-tailed distribution and thresholded at *p* ≤ 0.01 versus the control.

## 3. Results

### 3.1. Cinnamaldehyde and Curcumin Induce Post-Translational Modifications of Akt2 

**Figure 1:**
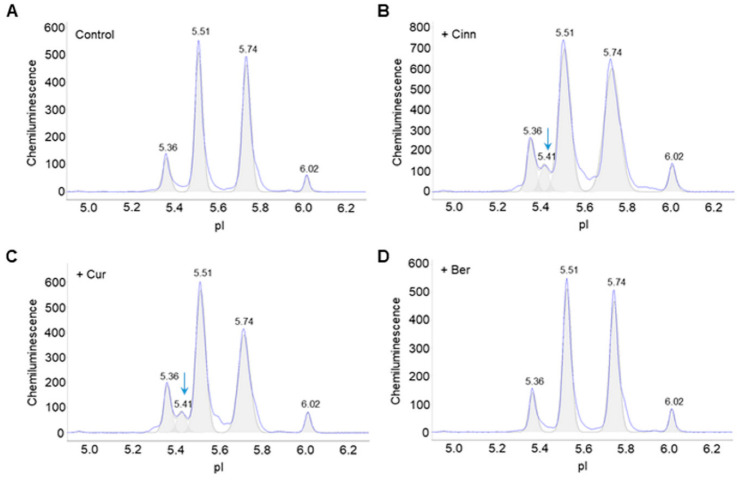
Cinnamaldehyde and curcumin induce changes to Akt2 post-translational modifications. Distribution of Akt2 as a function of isoelectric points in (**A**) control untreated preadipocytes, or (**B**–**D**) preadipocytes treated with (**B**) cinnamaldehyde, (**C**) curcumin, or (**D**) berberine. Arrows point to the appearance of new peaks following treatment versus untreated control.

The post-translational modification profiles of Akt2 in primary human omental preadipocytes were examined using capillary isoelectric focusing (cIEF) immunoassays. cIEF immunoassays are ultrasensitive and multiplexed methods that permit rapid detection of changes in the post-translational modifications of signaling proteins in response to extracellular stimulations [21,22,23,24,25,26,27,28,29,30,31,32,33]. Briefly, cIEF immunoassays separated Akt2 isoforms in total cell extracts by their isoelectric points in capillaries. The positions of Akt2 isoforms were stabilized via UV-irradiated crosslinking. Primary antibodies against Akt2 or p-Akt2 (S474) and secondary antibodies linked to horseradish peroxidase were sequentially introduced. Following the introduction of chemiluminescent substrates, the distribution of Akt2 isoforms as a function of isoelectric points was detected and presented graphically (Figure 1A). Treatment of preadipocytes with either cinnamaldehyde or curcumin induced the appearance of an additional Akt2 isoform at pI 5.41 compared to untreated control (Figure 1B,C). In contrast, treatment of preadipocytes with berberine had no effect on the distribution of Akt2 isoforms (Figure 1D). 

### 3.2. Cinnamaldehyde and Curcumin Enhance Insulin-Stimulated Activation of Akt2

**Figure 2:**
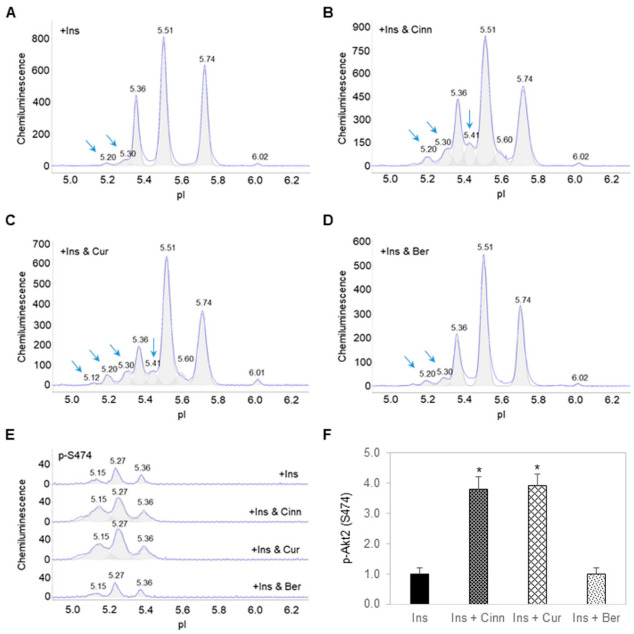
Cinnamaldehyde and curcumin enhance insulin-stimulated activation of Akt2. (**A**–**D**) Distribution of Akt2 as a function of isoelectric points in preadipocytes treated with (**A**) insulin alone, (**B**) insulin and cinnamaldehyde, (**C**) insulin and curcumin, or (**D**) insulin and berberine. (**E**) Distribution of p-Akt2 (S474) as a function of isoelectric points in preadipocytes treated with insulin alone (top electropherogram), insulin and cinnamaldehyde (second electropherogram), insulin and curcumin (third electropherogram), or insulin and berberine (bottom electropherogram). (**F**) Relative abundance of p-Akt2 (S474) as a function of treatment condition. Error bars are standard deviations across nine repeated measurements. Asterisk indicates *p*-value ≤ 0.01 versus treatment with insulin alone.

Treatment of preadipocytes with insulin-induced the appearance of additional Akt2 isoforms at low pI values of 5.20 and 5.30 (Figure 2A). Interestingly, treatment of preadipocytes with insulin together with either cinnamaldehyde (Figure 2B) or curcumin (Figure 2C) increased the magnitude of Akt2 isoforms at pI 5.20 and 5.30 versus treatment with insulin alone. The presence of cinnamaldehyde or curcumin consistently induced the appearance of a peak at pI 5.41 on the Akt2 electropherograms. In contrast, treatment of preadipocytes with insulin and berberine had no effect on the Akt2 electropherogram versus treatment with insulin alone (Figure 2D). The abundance of p-Akt2 (S474), which is required for Akt2 activation, increased by nearly four folds in preadipocytes treated with insulin together with cinnamaldehyde or curcumin versus treatment with insulin alone (Figure 2E,F). Expectedly, treatment of preadipocytes with insulin and berberine had no effect on the abundance of p-Akt2 (S474) versus treatment with insulin alone.

### 3.3. Additive Effects of Cinnamaldehyde and Curcumin on Akt2 Activation and Glucose Transport

**Figure 3:**
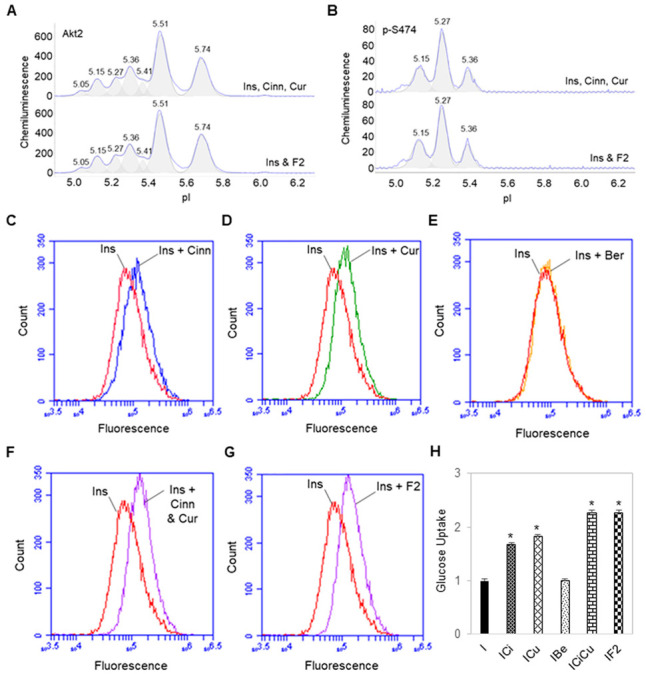
Additive effects of cinnamaldehyde and curcumin on Akt2 activation and glucose transport. (**A**,**B**) Distribution of Akt2 (**A**) and p-Akt2 (S474) (**B**) as a function of isoelectric points in preadipocytes treated with insulin (Ins), cinnamaldehyde (Cinn), and curcumin (Cur) (top electropherogram) or insulin, cinnamaldehyde, curcumin, and berberine (Ber) (bottom electropherogram). (**C**–**G**) 2-NBDG fluorescence in preadipocytes treated with insulin alone (red line) versus treated with (**C**) insulin and cinnamaldehyde (blue line), (**D**) insulin and curcumin (green line), (**E**) insulin and berberine (orange line), (**F**) insulin, cinnamaldehyde, and curcumin (purple line), or (**G**) insulin and F2 (cinnamaldehyde, curcumin, and berberine) (purple line). (**H**) relative abundance of 2-NBDG fluorescence in preadipocytes as a function of treatment condition. I, insulin; ICi, insulin and cinnamaldehyde; ICu, insulin and curcumin; IBe, insulin and berberine; ICiCu, insulin, cinnamaldehyde, and curcumin; IF2, insulin, cinnamaldehyde, curcumin, and berberine. Asterisk indicates *p*-value ≤ 0.01 versus treatment with insulin alone.

Treatment of preadipocytes with insulin together with both cinnamaldehyde and curcumin further enhanced insulin-stimulated activation of Akt2. On average, treatment of preadipocytes with insulin together with both cinnamaldehyde and curcumin increased the abundance of p-Akt2 (S474) by two folds versus treatment with either insulin and cinnamaldehyde or insulin and curcumin, or by approximately seven folds versus treatment with insulin alone (Figure 3A,B). Treatment of preadipocytes with insulin and a composition of phytonutrients comprising berberine, cinnamaldehyde, and curcumin, or F2 composition, had the same effect on Akt2 activation versus treatment with insulin, cinnamaldehyde, and curcumin. In other words, the presence of berberine did not interfere with the additive effects of cinnamaldehyde and curcumin on Akt2 activation. 

Cinnamaldehyde and curcumin also enhanced insulin-stimulated glucose uptake by preadipocytes. Treatment of preadipocytes with insulin together with either cinnamaldehyde or curcumin increased glucose uptake by approximately 70% versus treatment with insulin alone (Figure 3C,D). Treatment of preadipocytes with insulin and berberine had no effect on glucose uptake versus treatment with insulin alone (Figure 3E). Treatment of preadipocytes with insulin together with both cinnamaldehyde and curcumin increased glucose uptake by approximately 140% versus treatment with insulin alone (Figure 3F). Treatment of preadipocytes with insulin and F2 composition had the same effect on glucose uptake versus treatment with insulin together with both cinnamaldehyde and curcumin (Figure 3G). Again, the presence of berberine did not interfere with the additive effects of cinnamaldehyde and curcumin on glucose uptake (Figure 3H).

### 3.4. Berberine Inhibits Fat Cell Differentiation

**Figure 4:**
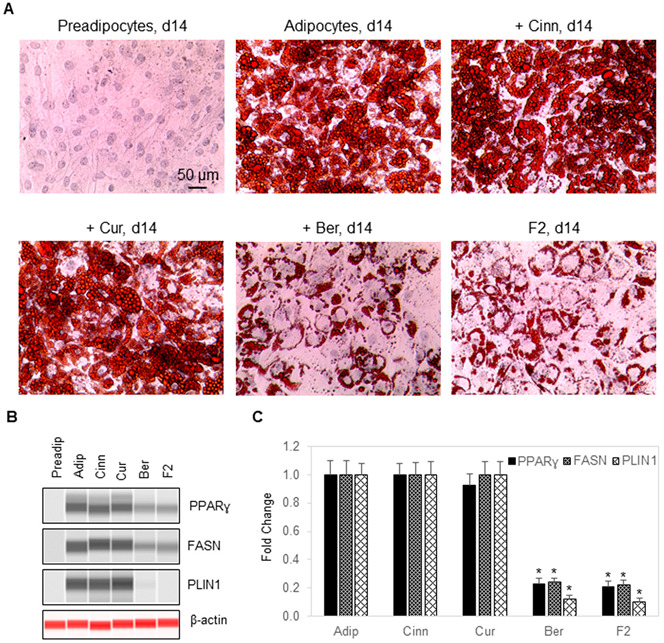
Berberine inhibits fat cell differentiation. (**A**) Hematoxylin, eosin, and Oil Red O staining of undifferentiated preadipocytes (first panel, upper row), differentiated adipocytes (second panel, upper row), differentiated adipocytes treated with cinnamaldehyde (third panel, upper row), differentiated adipocytes treated with curcumin (first panel, lower row), differentiated adipocytes treated with berberine (second panel, lower row), and differentiated adipocytes treated with an F2 composition comprising cinnamaldehyde, curcumin, and berberine (third panel, lower row). Hematoxylin, eosin, and Oil Red O staining was performed on day 14 post-differentiation. (**B**) Capillary Western immunoassays to evaluate the expression of adipogenic biomarkers PPARɣ (first row), FASN (second row), and PLIN1 (third row) in undifferentiated preadipocytes (first column), differentiating adipocytes (second column), differentiating adipocytes treated with cinnamaldehyde (third column), differentiating adipocytes treated with curcumin (fourth column), differentiating adipocytes treated with berberine (fifth column), and differentiating adipocytes treated with an F2 composition comprising berberine, cinnamaldehyde, and curcumin (sixth column). Total cell extracts used for CW immunoassays were collected on day six post-differentiation. β-actin (fourth row) served as a loading control. (**C**) Relative abundance of adipogenesis biomarkers PPARɣ, FASN, and PLIN1 as a function of treatment condition. Error bars indicate standard deviations across six repeated measurements per experimental condition. Asterisk indicates a statistical significance of *p*-value ≤ 0.01 versus differentiating adipocytes.

Treatment of preadipocytes with an adipogenesis cocktail comprising insulin, dexamethasone, IBMX, and rosiglitazone induced their differentiation into adipocytes [34,35]. The fat cell fate commitment was accompanied by intracellular lipid droplet accumulation and increased expression of adipogenic proteins, such as peroxisome proliferator-activated receptor ɣ (PPARɣ), fatty acid synthase (FASN), and perilipin 1 (PLIN1) [36]. Intracellular lipid droplets stained brightly with Oil Red O and expression of adipogenic proteins were measurable using capillary Western immunoassays (Figure 4A,B). Treatment with cinnamaldehyde or curcumin during fat cell differentiation had no impact on intracellular lipid droplet accumulation or expression level of PPARɣ, FASN, and PLIN1. In contrast, treatment with berberine strongly suppressed intracellular lipid droplet accumulation and expression levels of PPARɣ, FASN, and PLIN1 (Figure 4C). Berberine was primarily responsible for the anti-adipogenic effects of F2 composition.

### 3.5. Dietary Supplement with F2 Composition Prevents Weight Gain 

**Figure 5:**
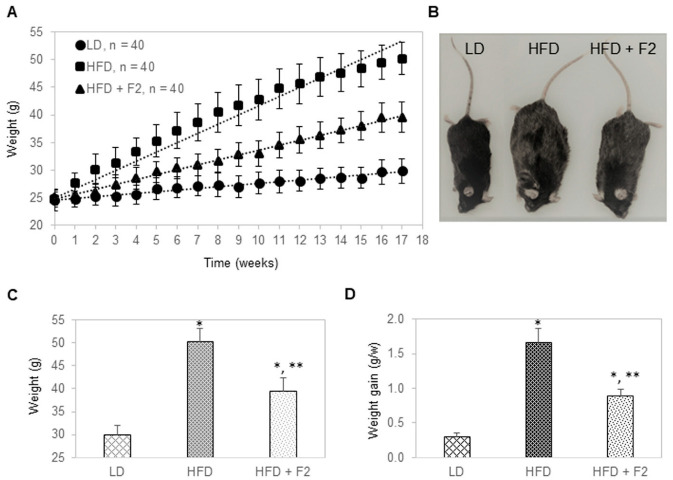
F2 composition reduces weight gain in diet-induced obesity mice. (**A**) Average body weight as a function of time on specified diets of three groups of mice: Lean diet, high-fat diet, and high-fat diet supplemented with F2. (**B**) A representative photo of mice from each diet group in week 17. (**C**) Average body weight in week 17 as a function of mice group on specified diets. LD: Lean diet; HFD: High-fat diet; HFD + F2: High-fat diet supplemented with F2. (**D**) Average rate of weight gain in grams per week (g/w) as a function of mice group on specified diets. The error bars indicate the standard deviations of 40 mice per animal group. Single asterisk (*) indicates a statistical significance of *p*-value ≤ 0.01 versus the lean diet group. Double asterisk (**) indicates a statistical significance of *p*-value ≤ 0.01 versus the high-fat diet group.

Next, the effects of F2 composition on glycemic and weight management were evaluated in a diet-induced obesity murine model. Mice were divided into three groups: A control group of 40 mice fed with a lean diet, a diet-induced obesity group of 40 mice fed with a high-fat diet, and an experimental group of 40 mice fed with a high-fat diet supplemented with F2. F2 was supplemented to ground pellets at 0.1% by weight, leading to an approximately daily dose of 200 mg/kg. All mice were placed on their respective diets for 17 weeks. All mice were male, approximately 10 weeks old, and had body weights of approximately 25 g at the start. Mice in the control group gained weight at a steady rate of 0.3 g per week and reached an average of 30 g in body weight after 17 weeks (Figure 5A–D). Mice in the diet-induced obesity group gained weight rapidly at a rate of 1.7 g per week and reached an average of 50 g in body weight after 17 weeks. Interestingly, mice in the experimental group gained weight at a rate of 0.9 g per week and reached an average of 40 g in body weight after 17 weeks. On average, a dietary supplement with F2 composition reduced weight gain of diet-induced obesity mice by approximately 50%. 

### 3.6. Dietary Supplement with F2 Composition Improves Glucose Tolerance and Reduces HbA1c

**Figure 6:**
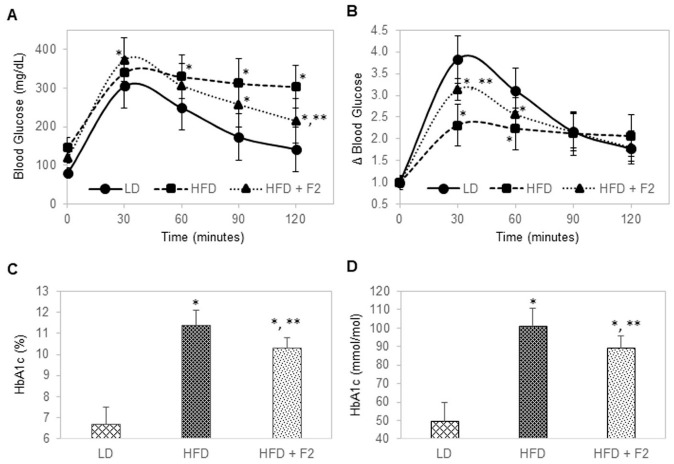
F2 composition improves glucose tolerance and reduces HbA1c in diet-induced obesity mice. (**A**) Blood glucose level as a function of time post-injection. (**B**) Fold change in blood glucose level as a function of time post-injection. (**C**) HbA1c (%) and (**D**) HbA1c (mmol/mol) as a function of animal groups. LD: Lean diet; HFD: High-fat diet; HFD + F2: High-fat diet supplemented with F2. The error bars indicate the standard deviations of 40 mice per animal group. Single asterisk (*) indicates a statistical significance of *p*-value ≤ 0.01 versus the lean diet group. Double asterisk (**) indicates a statistical significance of *p*-value ≤ 0.01 versus the high-fat diet group.

In week 16, glucose tolerance tests were performed for all mice after 16 h of overnight fasting (Figure 6A,B). Fasting blood glucose levels were 80, 147, and 119 mg/dL for mice of the control group (LD), diet-induced obesity group (HFD), and experimental group (HFD + F2), respectively (Figure 6A). Following intraperitoneal injection of 20% glucose at 2 g of glucose per kilogram of body mass, blood samples were collected via the tail veins at 0, 30, 60, 90, and 120 min and measured for blood glucose levels. Mice of the control group were able to dynamically regulate blood glucose level, where blood glucose increased by nearly four folds, peaked at around 30 min post-injection, and steadily declined to less than two folds higher than the baseline blood glucose level at 120 min post-injection (Figure 6B). In contrast, mice of the diet-induced obesity group were unable to regulate blood glucose levels, where blood glucose increased by 2.3 folds at 30 min post-injection and stayed elevated at above 2.0 folds higher than the baseline blood glucose level at 120 min post-injection. Interestingly, mice of the experimental group had improved control of blood glucose level versus mice of the diet-induced obesity group. Following glucose injection, blood glucose increased by more than three folds at 30 min post-injection, and steadily declined to less than two folds higher than the baseline blood glucose level at 120 min post-injection. Furthermore, HbA1c levels measured in terminally collected blood samples in week 17 were 6.7% or 50 mmol/mol for mice of the control group, 11.4% or 101 mmol/mol for mice of the diet-induced obesity group, and 10.3% or 89 mmol/mol for mice of the experimental group (Figure 6C,D).

### 3.7. Dietary Supplement with F2 Composition Reduces Blood Lipids

**Figure 7:**
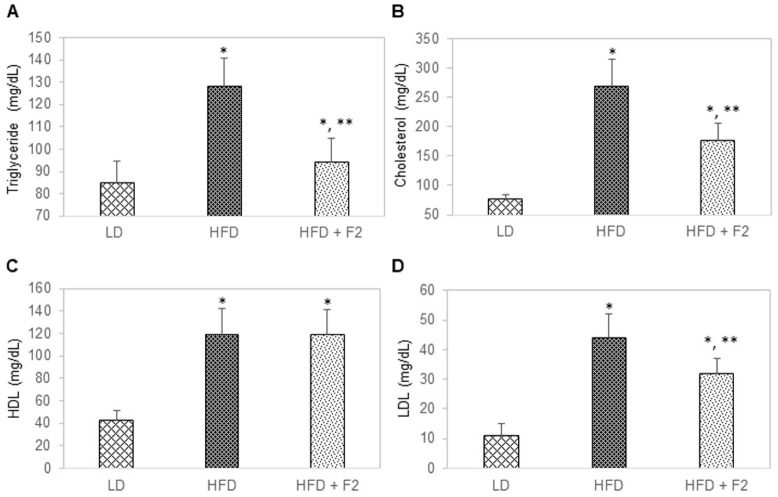
F2 composition reduces blood lipids in diet-induced obesity mice. (**A**) Triglyceride, (**B**) cholesterol, (**C**) high-density lipoprotein (HDL), and (**D**) low-density lipoprotein (LDL) as a function of animal group on specified diets. LD: Lean diet; HFD: High-fat diet; HFD + F2: High-fat diet supplemented with F2. Blood samples terminally collected after 17 weeks on specified diets were used for measurement. The error bars indicate the standard deviations of 40 mice per animal group. Single asterisk (*) indicates a statistical significance of *p*-value ≤ 0.01 versus the lean diet group. Double asterisk (**) indicates a statistical significance of *p*-value ≤ 0.01 versus the high-fat diet group.

In week 17, blood and tissue samples were terminally collected from all mice and measured for triglyceride, total cholesterol, high-density lipoprotein (HDL) cholesterol, and low-density lipoprotein (LDL) cholesterol. The average triglyceride levels were 85, 128, and 94 mg/dL for mice of the control group, diet-induced obesity group, and experimental group, respectively (Figure 7A). The average total cholesterol levels were 77, 269, and 176 mg/dL for mice of the control group, diet-induced obesity group, and experimental group, respectively (Figure 7B). The average HDL cholesterol levels were 43, 119, and 119 mg/dL for mice of the control group, diet-induced obesity group, and experimental group, respectively (Figure 7C). The average LDL cholesterol levels were 11, 44, and 32 mg/dL for mice of the control group, diet-induced obesity group, and experimental group, respectively (Figure 7D).

### 3.8. Dietary Supplement with F2 Composition Reduces Visceral Adiposity and Liver Steatosis

**Figure 8:**
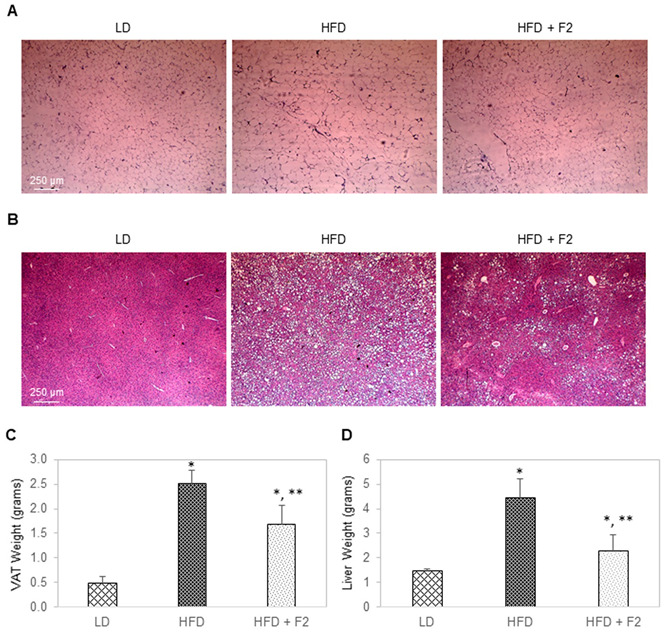
F2 composition reduces visceral adiposity and liver steatosis in diet-induced obesity mice. H&E histology of (**A**) visceral adipose tissues and (**B**) liver tissues collected from three animal groups on specified diets. (**C**) Visceral adipose tissue weight and (**D**) liver tissue weight as a function of animal groups on specified diets. LD: Lean diet; HFD: High-fat diet; HFD + F2: High-fat diet supplemented with F2. Visceral adipose tissues and liver tissues were terminally collected after 17 weeks on specified diets. The error bars indicate the standard deviations of 40 mice per animal group. Single asterisk (*) indicates a statistical significance of *p*-value ≤ 0.01 versus the lean diet group. Double asterisk (**) indicates a statistical significance of *p*-value ≤ 0.01 versus the high-fat diet group.

Hematoxylin and eosin (H&E) histology was performed on terminally collected liver and visceral adipose tissues (Figure 8A,B). On average, the diameters of visceral adipocytes of mice in the diet-induced obesity group and experimental group were two folds and 1.5 folds higher than the control group, respectively (Figure 8A). Consistently, the average weight of visceral adipose tissues was 0.5, 2.5, and 1.7 g for mice of the control group, diet-induced obesity group, and experimental group, respectively (Figure 8C). Furthermore, liver steatosis was the most severe in mice of the diet-induced obesity group and substantially reduced in mice of the experimental group (Figure 8B). On average, the liver weights were approximately 1.5, 4.5, and 2.3 g for mice of the control group, diet-induced obesity group, and experimental group, respectively (Figure 8D).

## 4. Discussion

In this study, a rational composition of phytonutrients (F2) for glycemic and weight management is presented. F2 comprises insulin-sensitizing phytonutrients, cinnamaldehyde and curcumin, and an anti-adipogenic phytonutrient, berberine. In primary human omental preadipocytes, cinnamaldehyde, and curcumin enhance insulin-stimulated activation of Akt2 and glucose uptake. Additionally, berberine inhibits fat cell differentiation. F2 promotes glucose uptake without activating the de novo fatty acid synthesis pathway in preadipocytes. In a diet-induced obesity animal model, a dietary supplement with F2 prevents weight gain, improves glucose tolerance, and reduces HbA1c, blood lipids, visceral adiposity, and liver steatosis. Collectively, a dietary supplement with F2 protects against pre-diabetic conditions in diet-induced obesity mice.

There are clear benefits to the combination versus individual phytonutrients for glycemic and weight management. For example, cinnamaldehyde and curcumin exhibit additive effects on the promotion of insulin-stimulated activation of Akt2 and glucose uptake in preadipocytes. Cinnamaldehyde and curcumin are known anti-adipogenic phytonutrients with effective concentrations of 40 and 20 µM in cell cultures, respectively [37,38]. However, their anti-adipogenicity diminishes quickly at lower concentrations and becomes completely ineffective at 10 and 4 µM for cinnamaldehyde and curcumin, respectively. On the other hand, berberine is a strong anti-adipogenic phytonutrient even at a low concentration of 2 µM [39]. The combination of cinnamaldehyde, curcumin, and berberine provides an effective means to improve insulin sensitivity without increasing adiposity.

Recently elucidated mechanisms of action of cinnamaldehyde, curcumin, and berberine support their complementarity. Cinnamaldehyde and curcumin are known inhibitors of protein serine/threonine phosphatases and protein tyrosine phosphatases [40,41]. Treatment of preadipocytes with cinnamaldehyde or curcumin increases phosphorylation of Akt2 at T450 and Y475, which primes Akt2 for subsequent activation via insulin-stimulated phosphorylation at S474 [17]. Cinnamaldehyde and curcumin are insulin sensitizers that act directly on the insulin signaling pathway. In contrast, berberine interacts with a potassium voltage-gated channel of pancreatic β-cells and promotes insulin secretion [42]. Berberine is useful for the management of diabetes by acting as an insulin secretagogue. Furthermore, berberine is a known inhibitor of adipogenesis, although the precise mechanism of action has not been delineated [20]. The current literature and data presented herein indicate that there is no known antagonism between cinnamaldehyde, curcumin, and berberine.

Bioavailability remains an insurmountable challenge for nutrition intervention for metabolic diseases. Like most phytonutrients, cinnamaldehyde, curcumin, and berberine are poorly soluble in aqueous solutions [43]. Their low plasma and tissue levels are due to poor absorption, rapid metabolism, and rapid systemic elimination [13,44,45,46]. Fortunately, they are natural ingredients that are safe and well-tolerated for human consumption at daily doses exceeding 20 mg/kg. Numerous strategies have been pursued to increase their bioavailability with limited success [47,48,49]. Alternately, rational combinations of phytonutrients based on their functional complementarity provide a means to reach intended biological effects with lower doses, which are achievable with natural bioavailability.

Most significantly, the combination strategy described herein is extensible to many phytonutrients with reported anti-obesity and anti-diabetic properties [10,50,51]. For example, numerous phytonutrients are known inhibitors of protein serine/threonine phosphatases and/or protein tyrosine phosphatases [52,53], whose combinations can improve insulin sensitivity in a manner comparable to that of cinnamaldehyde and curcumin combination. Like berberine, numerous phytonutrients exert anti-obesity and anti-diabetes effects independent of the insulin signaling pathway [10]. Furthermore, many phytonutrients have higher bioavailability compared to cinnamaldehyde, curcumin, and berberine, which render them more suitable for nutrition intervention [43]. Combining phytonutrients with functional complementarity provides a rational platform for the discovery of novel therapeutics for glycemic and weight management.

## 5. Conclusions

A composition of phytonutrients comprising berberine, cinnamaldehyde, and curcumin was effective in improving insulin sensitivity without increasing adiposity in a diet-induced obesity murine model. Future clinical assessment is necessary to evaluate safety and efficacy of the composition of phytonutrients comprising berberine, cinnamaldehyde, and curcumin for glycemic and weight management.

## Data Availability

All data supporting the findings of this study are available from the corresponding author upon reasonable request.
